# Doctor's Heroism

**Published:** 1920-06-05

**Authors:** 


### Doctor's Heroism.

Among the many brave efforts at rescue which were made during the terrible scenes caused by the flooding of Louth, that of Dr. W. J. Higgins is prominent. He had been called to a confinement in the river-valley just before the flood came, and he was in the upper floor of a small cottage with the woman and the nurse. When the water entered the cottage, the husband ran upstairs into the bedroom, where the water soon rose within a few inches of the bed. The husband got out of the window to fix a ladder as a means of escape, if necessary, but the rush of water was too strong for him and he soon got into difficulties. Dr. Higgins saw this, and jumped through the window into the water after the man. They succeeded in getting hold of a wire used as a clothes-line, and the doctor then swam to a roof and obtained a ladder, which he projected so that he was able to get the man to safety. Dr. Higgins then swam back to the open window of his patient's room, after the flood had begun to subside, brought the woman through her confinement, and later on managed to conduct her and the child to hospital, where they are both doing well. He returned to the work of rescue, is reported to have brought five children to safety, and he carried on until late into the night. Another medical man, Dr Laughton Smith, worked continuously at rescue work, going out in a small canoe into the rushing water and bringing to safety some women and children precariously situated in midstream.

